# Extra Purified Exosomes from Human Placenta Contain an Unpredictable Small Number of Different Major Proteins

**DOI:** 10.3390/ijms20102434

**Published:** 2019-05-16

**Authors:** Evgeniya E. Burkova, Alina E. Grigor’eva, Dmitrii V. Bulgakov, Pavel S. Dmitrenok, Valentin V. Vlassov, Elena I. Ryabchikova, Sergey E. Sedykh, Georgy A. Nevinsky

**Affiliations:** 1 SB RAS Institute of Chemical Biology and Fundamental Medicine, 8 Lavrentiev Ave., 630090 Novosibirsk, Russia; jorochka24@mail.ru (E.E.B.), feabelit@mail.ru (A.E.G.); valentin.vlassov@niboch.nsc.ru (V.V.V.); lenryab@yandex.ru (E.I.R.); sirozha@gmail.com (S.E.S.); 2 Federal Scientific Center of the East Asia Terrestrial Biodiversity, Far Eastern Branch of Russian Academy of Sciences, 690022 Vladivostok, Russia; bulgakov-dv@mail.ru; 3 G. B. Elyakov Pacific Institute of Bioorganic Chemistry FEB RAS, 159 100 let Vladivostoku Ave., 690022 Vladivostok, Russia; paveldmt@piboc.dvo.ru

**Keywords:** human placenta, exosomes purification, proteins separation, proteins identification

## Abstract

Exosomes are nanovesicles (30–100 nm) containing various RNAs and different proteins. Exosomes are important in intracellular communication, immune function, etc. Exosomes from different sources including placenta were mainly obtained by different types of centrifugation and ultracentrifugations and were reported to contain from a few dozen to thousands of different proteins. First crude exosome preparations from four placentas (normal pregnancy) were obtained here using several standard centrifugations but then were additionally purified by gel filtration on Sepharose 4B. Individual preparations demonstrated different gel filtration profiles showing good or bad separation of exosome peaks from two peaks of impurity proteins and their complexes. According to electron microscopy, exosomes before gel filtration contain vesicles of different size, ring-shaped structures forming by ferritin and clusters of aggregated proteins and their complexes. After filtration through 220 nm filters and gel filtration exosomes display typically for exosome morphology and size (30–100 nm) and do not contain visible protein admixtures. Identification of exosome proteins was carried out by MS and MS/MS MALDI mass spectrometry of proteins’ tryptic hydrolyzates after their SDS-PAGE and 2D electrophoresis. We have obtained unexpected results. Good, purified exosomes contained only 11–13 different proteins: CD9, CD81, CD-63, hemoglobin subunits, interleukin-1 receptor, annexin A1, annexin A2, annexin A5, cytoplasmic actin, alkaline phosphatase, serotransferin, and probably human serum albumin and immunoglobulins. We assume that a possible number of exosome proteins found previously using crude preparations may be very much overestimated. Our data may be important for study of biological functions of pure exosomes.

## 1. Introduction

Human placenta is a very highly specialized organ that connects mother and fetus organisms, and placental dysfunction could lead to catastrophic consequences for both. The villi of the placenta are bordered with syncytiotrophoblast cells, which form the hemato-placental barrier separating the mother and fetus blood circulations [1]. The placenta protects, nourishes and regulates the growth of the embryo [2,3,4]. Progress in study of pregnancy and placenta functioning may contribute to the development of transplantation methods; this requires a detailed study of the biochemical characteristics of mother and fetus.

Exosomes, which are vesicles with a diameter of 30–100 nm, have a unique molecular composition capable of implementing many functions, including cell-to-cell communication [5,6]. Signal transmission between cells is particularly important for the mother–fetus system, in which the placenta is the organ providing interaction between two organisms and preserving their individuality [1,2,4]. The transfer of molecular signals between the fetus and mother is still foggy, and the possible role of exosomes in this process was not studied.

It is known that exosomes are formed by invagination of the membrane of late endosomes, in which they accumulate, being subsequently released from cells [5,6]. In accordance with the modern classification, exosomes belong to the class of extracellular vesicles, which also includes microvesicles and apoptotic bodies. Exosomes were isolated from various biological fluids of humans and mammals, including milk [1,5,6,7,8,9,10,11,12,13,14].

Relevance of the study of placenta exosomes and their molecular composition is determined by the presence in placenta of a variety of factors, including biologically active RNA, DNA, proteins, peptides, antibodies and other components [1,2,3,4]. 

Exosome studies are carried out using preparations isolated from biological fluids by a wide range of methods, including sedimentation by centrifugation followed by ultracentrifugation as well as ultracentrifugation in a density gradient [15,16,17,18,19,20,21,22,23,24,25,26,27]; ultrafiltration through a filter with a pore size of 0.1–0.2 μm, and by other methods [25,26,27,28,29]. It should be noted that all these methods make it possible to obtain preparations enriched in exosomes, but not homogeneous pure exosome preparations, thus affecting the molecular composition of exosomes. In fact, a type of the method of exosome isolation leads to the high variability of the data, and the range of variations of the number of proteins identified in the exosomes amounting to hundreds of times in different publications [15,16,17,18,19,20,21,22,23,24,25,26,27,28,29]. 

A meta-analysis of 78 published databases of exosome revealed up to 797 proteins identified in exosome preparations [7] obtained from normal and cancer cells’ culture fluids, as well as biological fluids and tissues of various species, including humans, mice, rats, cows, flies, yeast, and even bacteria [13]. Data on the protein composition of placenta exosomes are scarce and vary greatly in different studies. Depending on the methods of protein analysis, several hundreds and even thousands of proteins were found in preparations of placental exosomes: 349 [30] and 1476 proteins [31].

In the proteomic analysis of human syncytiotrophoblast microvesicles, more than 400 proteins were identified [32]. Differences in expression of 25 proteins (integrins, annexins, and histones) were found in preeclampsia compared with healthy pregnant women. The results of the study [1] led to assumption that there may be both general and specific markers of exosomes from different sources.

According to the literature, exosomes of different origin may contain different amounts of proteins. For example, crude preparations of exosomes of dendritic cells can contain more than 150–200 different proteins [32,33,34]. An even more improbable result was obtained when analyzing proteins of the milk’s exosomes of cows, whose preparations were isolated by centrifugation and ultracentrifugation in a sucrose gradient [35]. As a result, 2107 proteins have been identified, which include all major protein markers of exosomes detected previously. The most represented among the proteins of the milk exosomes of the cow were the proteins of the membranes of the fat globules of milk: butyrophilin, xanthine oxidase, adipophilin, and lactahedrin [36].

Our experience shows that the methods used for the isolation of exosomes used by researchers allow for obtaining only preparations enriched in exosomes to one degree or another. Biological fluids contain supramolecular aggregates of proteins, which, when centrifuged and ultracentrifuged, are co-precipitated with exosomes. In addition, we recently showed that human milk and placenta contain very stable high molecular weight (~1000 kDa) multiprotein complexes comparable in size to exosomes [37,38]. In addition, some proteins can specifically or nonspecifically bind to the surface of exosomes or receptors embedded in their membrane. Taking into account all these factors, we believe that the reported in literature amount of proteins contained in exosomes can be greatly overestimated. In this regard, a critical review by E.D. Sverdlov could be noted, who believes that, in the case of exosomes, there is an incorrect overestimated quantitative assessment of their internal molecular components, which, in his figurative expression, “would certainly make Amedeo Avogadro cry” [39].

Recently, we performed a proteomic analysis of the exosomes of horse milk [40]. Preparations of exosomes, obtained using several conventional centrifugations and ultracentrifugations, contained not only exosomes (40–100 nm), but also many smaller and larger vesicles, as well as impurity proteins and their oligomeric complexes. After additional purification of exosomes from stable high-molecular complexes and other proteins using gel filtration, only 7–8 different major proteins were detected in the composition of the milk, depending on milk sample [40]. All other different proteins previously found in the preparations of cow and human milk exosomes were found in the second peak with a lower molecular weight eluted after the first peak of exosomes [40].

The objective of [41] was to attract the attention of researchers to the problem of contamination of exosome preparations. Exosome preparations were isolated by sequential centrifugation according to widely accepted schemes from various biological fluids. All preparations (more than 200) contained exosomes identified by immuno-electron microscopy for binding to antibodies against CD63 or CD9. In addition to the exosomes, all preparations contained low electron density contaminating structures that did not have a limiting membrane and, accordingly, were not exosomes (“non-vesicles”). Two main types of “non-vesicle” are noted: having a size of 20–40 nm and constituting 10–40% of all structures of exosomes having a size of 40–100 nm. The morphology of “non-vesicle” allowed them to be classified as intermediate and low density lipoproteins (20–40 nm) and also very low density (40–100 nm). Thus, exosome preparations without additional purification may contain impurities of a very different nature, including proteins and nucleic acids. Such crude exosome preparations were analyzed exactly for the content of proteins and nucleic acids in most of the published articles.

In this study, we analyzed the exosome proteins obtained from four human placentas using standard approaches of centrifugation and additionally purified by gel filtration. Gel filtration allowed separating a large number of proteins from exosome preparations, which were co-precipitated with exosomes during different procedures of centrifugation. Proteins of well-purified exosomes were separated using 1D and 2D electrophoresis and identified using MALDI MS and MS/MS spectrometry of their tryptic hydrolysates as in the case of milk of horses [40]. We obtained unexpected results: exosomes isolated from placentas contain only 10–12 major proteins.

## 2. Result

**Figure 1:**
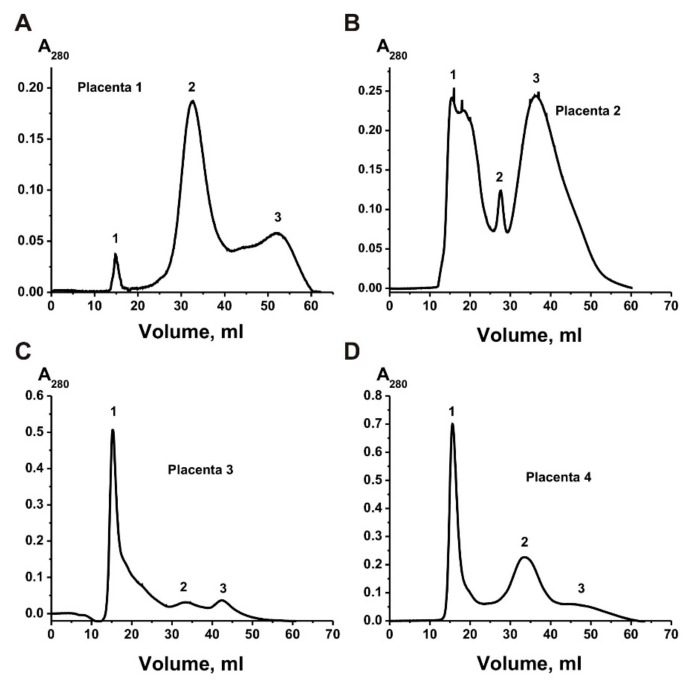
Four different profiles of four placenta crude exosome preparations (previously partially purified by several different centrifugations and filtration through filter 0.1 μm) purification by FPLC gel filtration on Sepharose 4B column: (—), absorbance at 280 nm (A_280_). Four different preparations of exosomes (**A**–**D**). One can see three peaks after gel-filtration (1–3).

By centrifugation and ultracentrifugation according to [14,15,16,17,18,19,20,21,22,23,24,25,26,27,28,34,35,36] with some changes [40], first, we obtained preparations enriched in exosomes from four human placentas. For additional purification of exosomes’ preparations, the obtained preparations were passed through filters (0.1–0.22 μm), and then gel filtered on a Sepharose 4B separating proteins of 60–20,000 kDa. Figure 1 shows four different gel filtration profiles—exosomes eluted at the first peak, which were separated from the two peaks of the impurity proteins. Four exosomes’ preparations demonstrate different ratios of the absorption (A_280_), corresponding to the first peaks of exosomes and two peaks of impurity proteins co-precipitated with them during different centrifugations.

**Figure 2:**
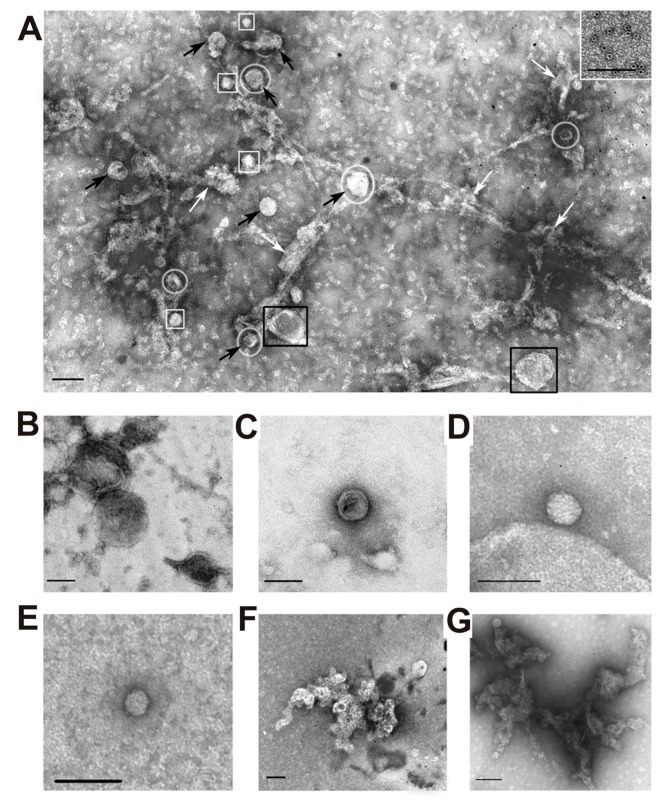
Transmission electron microscopy, negative contrast. General view of the preparation of placental vesicles before gel filtration: vesicles with a diameter of less than 100 nm (black arrows), vesicles larger than 100 nm (black squares), microparticles (white squares), amorphous protein aggregates (white arrows), structures that look related to protein associates are highlighted with white ovals (**A**). Large membrane structures with a diameter of more than 100 nm (**B**); vesicles with a size of less than 100 nm (**C**), non-vesicles with a size of more than 40 nm (**D**); non-vesicle size less than 40 nm (**E**), clusters of aggregated proteins (**F**–**G**); ferritin ring structures (10–14 nm) (inset in Figure A). The length of the scale line is 100 nm.

After each stage of the isolation, exosome preparations were analyzed using transmission electron microscopy. Before filtration, all preparations contained vesicles with a diameter less than 100 nm (black arrows) and vesicles with a diameter greater than 100 nm (black squares), microparticles (white squares), and amorphous protein aggregates (white arrows) (Figure 2A). Figure 2 also shows several separate specific structures of preparations enriched in exosomes: large membrane structures greater than 100 nm in size (B); vesicles with a diameter of less than 100 nm (C), microparticles having no a limiting membrane, more than 40 nm in size (microparticles of population II with a diameter of 40–60 nm) (G), as well as microparticles with a size less than 40 nm (microparticles of population I with a diameter of 20–40 nm) (D); clusters of aggregated proteins (E and G); and ferritin ring structures (10–14 nm) (inset to A).

**Figure 3:**
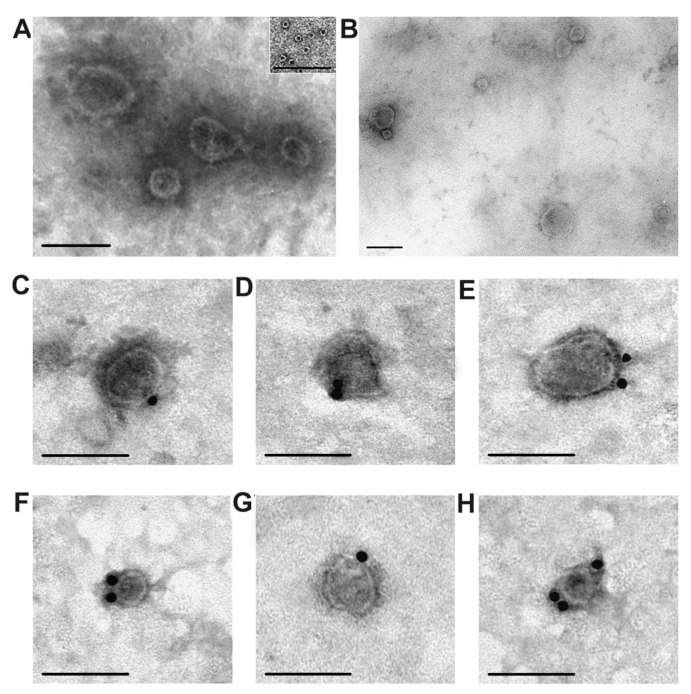
Transmission electron microscopy, negative contrast. Preparations of placental exosomes after filtration through a filter with pores of 100 nm and subsequent gel filtration (**A,B**). Vesicles (**A**) and ferritin ring structures (10–14 nm; inset in Figure A), visible protein clusters are absent. Exosomes purified by gel filtration, labeled with conjugates of gold nanoparticles with monoclonal antibodies against tetraspanin CD81 (**C**,**D**, and **E**) and against CD63 (**F**,**G**, and **H**). The length of the scale line is 100 nm.

After gel filtration, the preparations contained exosomes of various sizes (30–100 nm), as well as some ring supramolecular formations (10–14 nm), but did not contain visible amorphous protein material (Figure 3A,B). As a rule, exosomes must contain CD81 and/or CD63 [5]. We used a direct method; the vesicles purified by gel filtration contain CD81 and CD63; these marker proteins were labeled with antibodies against CD81 (Figure 3C,D) and CD63 (Figure 3E–G). Thus, purified placental vesicles comply corresponding to the requirements for typical exosomes in terms of morphology, size, and content of tetraspanins CD81 and CD63 on their surface.

**Figure 4:**
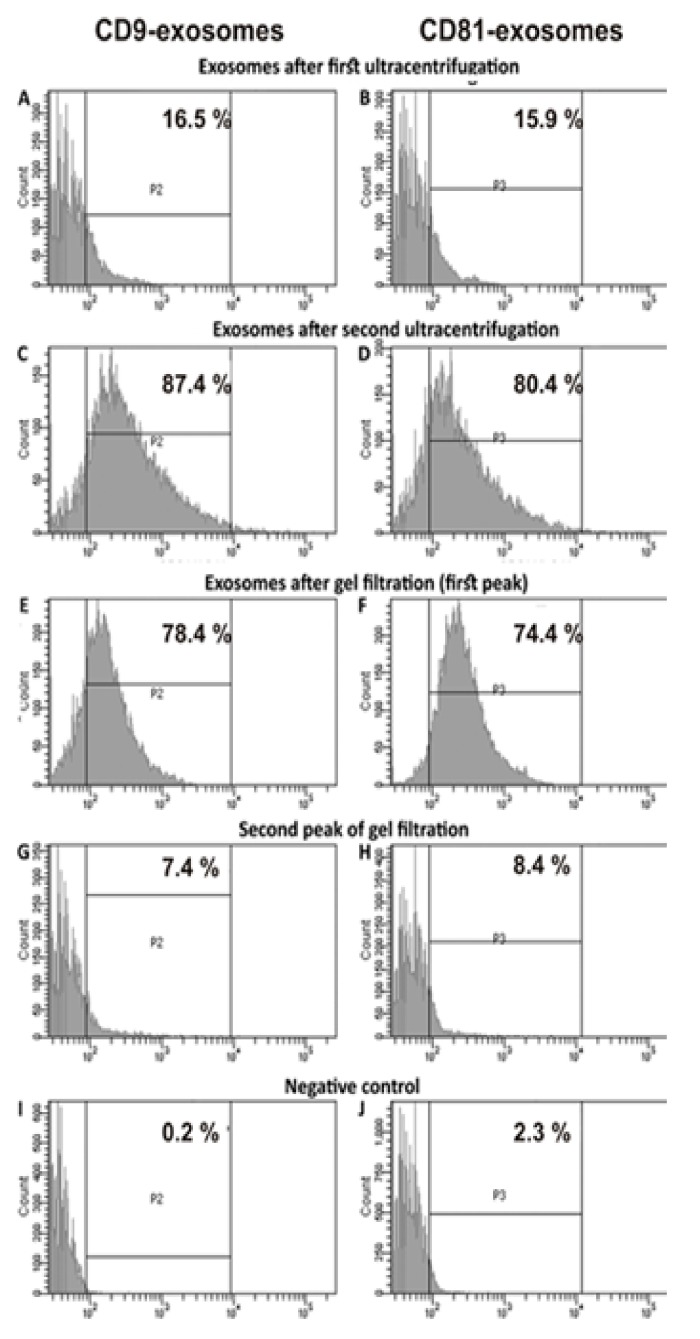
Flow cytometry of exosome preparations after two stages of ultracentrifugation and gel filtration. The relative amount of vesicles containing CD9 (left column of illustrations) and CD81 (right column of illustrations) are shown. All designations are given in panels (**A**–**J**).

**Figure 5:**
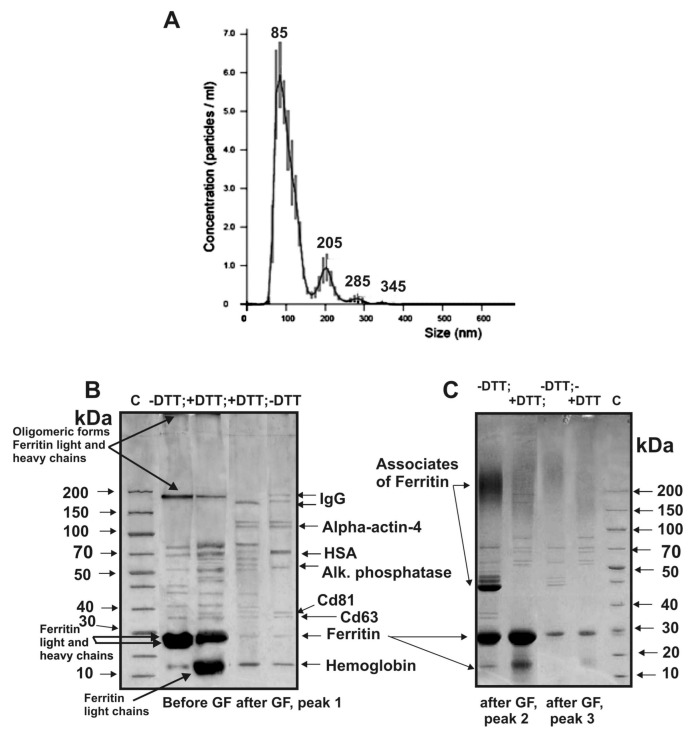
Nanoparticle tracking analysis of exosomes size after preparation gel filtration using Nanosight NS300 equipment (**A**). SDS-PAGE analysis of the intrinsic proteins of exosome preparation exo-1 after its filtration though filters 0.1 μm, but before FPLC gel filtration (**B**; two first lanes: –DTT and +DTT) as well proteins of exosome peak 1 after gel filtration (B; two second lanes: –DTT and +DTT). Analysis of impurity proteins corresponding to peaks 2 and 3 (Figure 1A) after gel filtration of exo-1 preparation (**C**; –DTT and +DTT lanes). Lanes C correspond to control proteins with known MMs (B and C). Identification of proteins was performed using MALDI mass MS and MS/MS data of proteins hydrolysates corresponding to the protein bands after SDS-PAGE; identified proteins are listed in B and C.

The relative amount of vesicles containing CD9 and CD81 was estimated using flow cytometry after first, second ultracentrifugation, and gel filtration (Figure 4). Placenta vesicles after gel filtration contain 78.4 ± 4.0 % of CD9-vesicles and 74.4 ± 4.0 % of CD81-particles (Figure 4E,F). The yield of particles after gel filtration was relatively high, ~90%–93%. Thus, it is obvious that gel filtration does not lead to the loss of the main part of exosomes. The content of vesicles of different sizes after gel filtration was evaluated using the nanotrack analysis (Figure 5A). Average size of the vesicles found by nanotrack analysis estimating hydrodynamic size of particles (and unable to distinguish individual particles and their associates) was remarkably higher than that in case of electron microscopy. Nevertheless, the average size of the main part of the vesicles (84 ± 5 nm; 86% ± 3%) is quite consistent with electron microscopy data on the presence in preparations of particles mainly with a size of from 40 to 100 nm.

Using SDS-PAGE, we analyzed the proteins of three peaks obtained by gel filtration. Figure 5B shows typical results of SDS-PAGE of the exo-1 corresponding to the first exosome preparation; the peak of exosomes is well-separated from the second and third peaks of protein impurities. Electron microscopy revealed ring supramolecular structures formed by ferritin (10–14 nm) in exosome preparations before and even after gel filtration (Figure 3A). According to results of SDS-PAGE before gel filtration, the light and heavy chains of ferritin are major proteins of the exo-1 (Figure 5C) and three other preparations.

In the absence of dithiothreitol (DTT), a part of ferritin corresponds to monomers with MMs ~23–25 kDa, its oligomeric forms of ~180 kDa, and ferritin–proteins complexes that are not included in the gel (Figure 5C, lane –DTT; before gel filtration). Treatment of exo-1 with DTT leads to partial destruction of the oligomeric forms and to an increase of protein in bands corresponding to light chains of ferritin (Figure 5C, +DTT). Peaks 2 and 3 before the treatment of exosomes’ preparations with DTT contain mainly oligomeric forms of ferritin (Figure 5C, –DTT). After treatment with DTT, exo-1 contains fewer ferritin oligomeric forms (Figure 5C, +DTT). Gel filtration leads to nearly complete disappearance of ferritin from exo-1 preparation (Figure 5B, –DTT). Similar results were obtained for three other exosome preparations before and after gel filtration.

The gel filtration of the exo-2 preparation weakly separated the peak of exosomes from the peak of proteins co-precipitated during the centrifugations (Figure 1B), and the resulting preparations of exosomes contained many different forms of ferritin even at the first peak corresponding to exosomes.

Eight major proteins were identified using MALDI mass spectrometry and MS/MS of tryptic protein hydrolysates corresponding the protein bands after one-dimensional SDS-PAGE of the proteins of first peak of exo-1 separated by the gel-filtration: hemoglobin, ferritin (monomer and oligomers), CD63, CD81, alkaline phosphatase, human serum albumin (HSA), alpha-actin-4 and IgGs (Figure 5B, −DTT, peak 1, Table 1).

After similar SDS-PAGE of the first peak of exo-2 eleven proteins were identified: ferritin (monomer and oligomers), hemoglobin, CD81, CD63, annexin, cytoplasmic actin, alkaline phosphatase, HSA, serotransferrin, alpha-actin-4, and IgGs.

**Figure 6:**
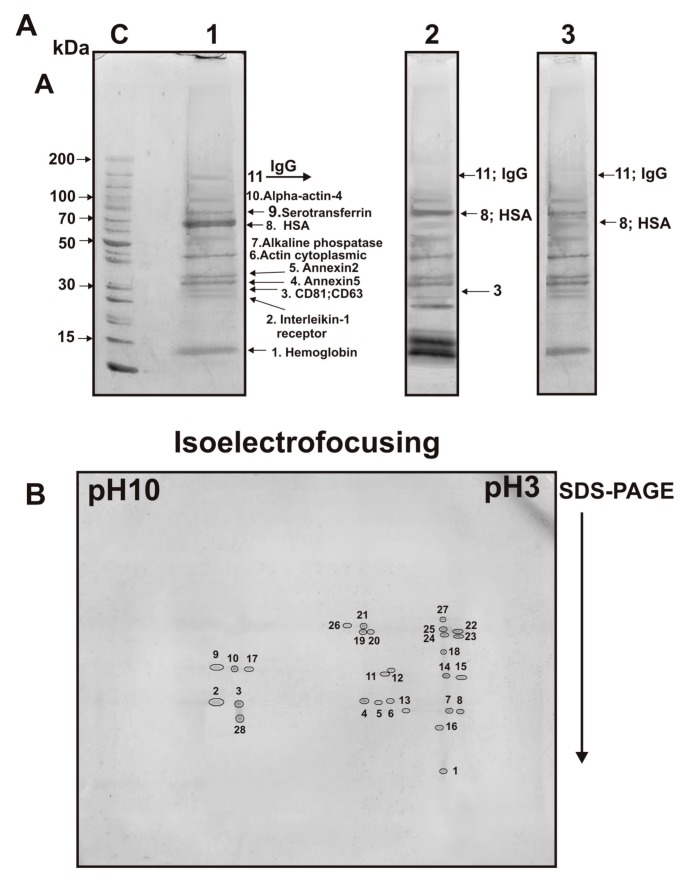
SDS-PAGE analysis of proteins of the exo-3 preparation after its gel filtration before (lane 1) and after treatment with dithiothreitol (lane 2), as well as after trypsin treatment (lane 3) (**A**). Molecular weight markers (lane C). After DTT treatment, the molecular weight of HSA increases due to the reduction of disulfide bonds (lane 2), and the band corresponding to IgG disappears due to the formation of free heavy and light chains. Treatment with trypsin leads to the hydrolysis of HSA, IgGs, CD63, and CD81, but not other proteins of this preparation (lane 3, A). Two-dimensional gel electrophoresis of exo-3 proteins after its isolation by gel filtration (**B**). The proteins were first separated using isoelectric focusing and then by SDS-PAGE. Twenty-eight protein spots stained with Coomassie R-250 correspond to: hemoglobin subunits (spot 1), annexin A2 (2, 3, 4, and 28), annexin A5 (5, 6, and 7), annexin A1 (8), actin cytoplasmic (9, 10, 11, 12, 13, 14, and 15), light (16) and heavy (17 and 18) chains of immunoglobulins, alkaline phosphatase (19, 20, 21, 22, and 23), HSA (24 and 25), and serotransferrin (26, 27).

For exo-3, only 11 proteins were identified (Table 1); it contained additionally two annexins: annexin5 and annexin2 (Figure 6A). Interestingly, in the case of exo-3, ferritin was almost absent (Figure 6A, track 1). Thus, ring ferritin supramolecular structures, identified in exosomes’ preparations by electron microscopy (Figure 3), in principle can be removed by gel filtration and therefore should be considered as a protein co-precipitated with exosomes during different centrifugations.

Next, we analyzed the proteins of the first peak obtained by gel filtration of the exo-3 preparation (Figure 1C) using 2D electrophoresis and identified 28 stained spots (Figure 6B) corresponding only to nine different proteins and their isoforms (Table 2). Interestingly, only one spot corresponded to the subunits of hemoglobin and annexin A1. The remaining spots corresponded to different isoforms of proteins (number of protein spots): annexin A2 (4), annexin A5 (3), cytoplasmic actin (7), alkaline phosphatase (5), HSA (2) and serotransferrin (2). Three protein spots were identified as light and heavy chains of immunoglobulins (Table 2). Thus, 2D electrophoresis allowed identification of only nine proteins. At the same time, three minor proteins CD81, CD63, and alpha-actin-4 were identified only after 1D electrophoresis; 2D electrophoresis allowed for identifying additionally annexin A1 (Table 2).

When analyzing proteins of the first peak of exo-4 by SDS-PAGE, eleven proteins were identified: different forms of ferritin, hemoglobin, CD81, CD63, annexin-2, annexin-5, cytoplasmic actin, alkaline phosphatase, HSA, alpha-actin-4, and IgGs.

Thus, four samples of exosomes contained several common proteins (the number of preparation in which this protein is identified): ferritin (3), hemoglobin (4), CD81 (4), CD63 (4), various annexins (4), cytoplasmic actin (4), alpha-actin-4 (3), alkaline phosphatase (4), HSA (4), serotransferrin (4), and IgGs (4). The data on the identification of all proteins using MS and MS/MS after electrophoretic analysis (1D and 2D) for four preparations of exosomes are presented in Supplementary Table S1.

However, it cannot be stated unequivocally that all identified proteins are internal proteins of exosomes. Some of them can co-precipitate due to their effective interaction with the surface of exosomes. Figure 2A demonstrates that some exosome particles look bound to proteins and their complexes. Therefore, before SDS-PAGE, we treated the exo-3 preparation with trypsin and chymotrypsin. As can be seen from Figure 6A (lane 3), after treatment with proteases, HSA, and immunoglobulins, as well as proteins CD81 and CD63, almost disappeared. The proteolysis of CD81 and CD63 can be easily explained by the fact that these proteins are presented on the surface of exosomes and so are easily accessible for proteases. HSA and immunoglobulins appear to be just co-precipitated with exosomes, or they are somehow strongly associated with the surface of the exosomes or with the exosome membrane proteins.

## 3. Discussion

Crude vesicle preparations (only different centrifugations) were first obtained from four placentas. They were very dirty and contained numerous structures of different vesicles with a size of ~30–300 nm, various proteins and their large associates (Figure 2). Filtration of these preparations through filters (100 nm), as well as purification by gel filtration, leads to removal of some types of vesicles as well as many co-precipitated proteins and their complexes (Figure 1 and Figure 3). Separated by gel filtration exosomes contain CD81 and CD63 (Figure 3). Thus, vesicles isolated from human placenta correspond to exosomes previously obtained from various sources [5] in terms of their morphology, size (30–100 nm), and content of CD81 and CD63 tetraspanins on their surface.

According to literature, exosomes from various biological fluids and tissues may contain from several tens to thousands of different proteins. However, the most studies of exosome preparations [1,5,6,7,8,9,10,11,12,13,14,15,16,17,18,19,20,21,22,23,24,25,26,27] obtained using various centrifugations contain a large number of very different impurities including different vesicles, structures having no membranes (“non-vesicles”), proteins and their associates (Figure 2). Gel filtration of such preparations leads to the separation of the main impurities. MALDI mass spectrometry analysis of placenta exosomes isolated by gel filtration identified only a few major proteins. However, a very large number of different proteins were detected in the second and third peak after gel filtration (Figure 1).

After 2D electrophoresis, 28 protein spots were found, which corresponded to only nine different proteins and their isoforms (Table 2). Overall, using two methods of electrophoretic analysis, twelve proteins were identified in four exosome preparations: CD81, CD63, hemoglobin subunits, annexin A1, annexin A2, annexin A5, cytoplasmic actin, alpha-actin-4, alkaline phosphatase, serotransferrin, HSA, immunoglobulins (Table 1 and Table 2). In addition, CD9 was revealed using flow cytometry (Figure 4).

Ferritin disappears after gel filtration and exosomes treatment with proteolytic enzymes. After treatment of exosome preparations with trypsin and chymotrypsin, the protein bands corresponding to HSA and immunoglobulins according to SDS-PAGE data almost completely disappeared. It cannot be excluded that HSA and immunoglobulins form relatively stable complexes with tetraspanins or interact directly with the surface of exosomes.

Thus, the question arises why we have detected much less proteins than has been previously found by other researchers [33,34]. Figure 1 shows that the relative absorption of the exosome peak at 280 nm is less than 7% compared with the second and third gel filtration peaks corresponding to co-precipitated proteins. Figure 2A shows that poorly purified preparations contain not only vesicles of various sizes, but also numerous proteins and their complexes. Previously published articles described exosomes obtained from placental trophoblast cells [34,35]. Therefore, it cannot be ruled out that placental cells during normal pregnancy and cultured cells can secrete exosomes containing different sets of proteins.

From our point of view, the actual number of proteins (several tens and thousands) that are directly included in the exosomes isolated from various sources can be greatly overestimated.

As it was mentioned above according to literature data, placental exosomes can contain frim several tens to thousands of different proteins [30,31]. For example, among many proteins of placental exosomes CD9, CD63, CD81 [30,31], placental alkaline phosphatase [10], pro-apoptotic FasL1–4 and TRAIL [9], the regulatory cytokine TGFb [10], syncytin-1 and syncytin-2 proteins [42] were found. In exosome preparations of female placenta, we found only some of these proteins: CD9, CD81, CD-63, and alkaline phosphatase. Since in published papers [9,10,30,31,42] exosomes were isolated by only various types of centrifugation, our data cannot be compared with them, and an open question remains: which of the discovered proteins are located directly in exosomes? In this regard, we should note the following data. Highly purified exosome preparations were obtained from human colon line DKO-1 by sequential combinations of three different methods, ultra-centrifugation, high-resolution density gradient fractionation, and direct immunoaffinity capture [43]. It was shown that these exosome preparations contain only fifteen major proteins: CD9, CD63, CD81, annexin A2, annexin V, TSG101, syntenin 1, ALIX, 14-3-3 zeta/delta, 14-3-3 epsilon, HSC70, EEF1-A1, aldolase A, enolase I, and LADH. We found six of these proteins in placental exosomes: CD9, CD81, CD-63, annexin A2, annexin A5. However, the content of different proteins in exosome preparations from different organs and cells can most probably vary greatly. For example, extra-purified exosomes from several different horse milk contained only five the same major proteins (CD9, CD81, CD63, beta-lactoglobulin, and lactadherin), while actin, butyrophilin, lactoferrin, and xanthine dehydrogenase were found only in some of them [40]. An even greater difference in the composition of proteins can be in the case of exosomes from various organs and cells.

The significance and function in the exosomes of the major proteins identified by us have not yet been elucidated, but all these proteins perform important functions in the organism of mammals [44,45,46,47,48]. For example, annexins have been shown to be directly involved in the formation of vesicles and the implementation of vesicular transport during exocytosis and endocytosis.

In this work, we analyzed the major proteins of the additionally purified exosomes from human placenta and showed that they contain a relatively small number of major proteins. It is possible that exosomes can also contain some minor proteins that cannot be detected using SDS-PAGE and MALDI mass spectrometry. Nevertheless, the identification of hundreds and thousands of proteins in the composition of the exosomes seems to us extremely overestimated. In addition, the diversity of proteins found in exosomes raises questions about whether proteins that co-precipitated with these vesicles, as well as possible intrinsic minor proteins of exosomes, have or do not have any important role in the biological functions of exosomes.

## 4. Materials and Methods

### 4.1. Materials

Reagents and sorbents including Sepharose 4B were obtained from Sigma (St. Louis, MO, USA). The blood sampling protocol conformed to the local human ethics committee guidelines (Approved 20 January 2015 by Ethics committee of Novosibirsk State Medical University, Novosibirsk, Russia; Institutional ethics committee specifically approved this study) in accordance with Helsinki ethics committee guidelines. All mothers gave written consent to present of their placentas for scientific purposes. The obstetricians/gynecologists provided us with anonymous placenta samples from normal pregnancy mothers (20–32 years old) having no history of autoimmune, rheumatologic, respiratory, cardiovascular, gastrointestinal, reproductive, or nervous system pathologies. These women gave birth to healthy children.

### 4.2. Placenta Extracts Preparation

Every fresh placenta was placed in a solution of 2.0 L of 1% sodium citrate immediately after birth to prevent the coagulation of the blood. For removal from placenta of blood, a solution of 1% sodium citrate containing 0.1% NaCl was passed under pressure through blood vessels of the placenta using several syringes (40 mL) connected with different thickness needles corresponding to all large, medium, and small blood vessels. Then, a fresh placenta (~400–500 g) was cut into small pieces, washed three times with buffer (20 mM Tris-HCl, pH 7.5, 125 mM KCl, 0.5 mM EDTA-NaOH, pH 7.5, and 0.5% sodium citrate) to remove remaining blood. Next, the crushed pieces of the placenta were homogenized in cold buffer (+4 °C; 425 mL) containing 250 mM sucrose, 20 mM Tris-HCl (pH 7.5), 125 mM KCl, 10 mM MgCl_2_, 0.5 mM EDTA (pH 7,5) and 0.5% sodium citrate. The homogenate of placenta was centrifuged at 26,000× *g* for 30 min (Beckman Coulter Avanti-J-301 centrifuge, JA-30.50Ti rotor, Brea, CA, USA), the precipitate was removed. The supernatant was dialyzed against H_2_O for 2 h, then against TBS buffer (20 mM Tris-HCl, pH 7.5, 0.15 M NaCl) for 12 h, and the dialyzed supernatant was used to isolate the exosomes.

### 4.3. Purification and Characterization of Vesicle Preparations

Placenta extracts preparations were obtained from total placentas. Supernatants were subjected to sequential centrifugation: twice at 10,000× *g* for 40 min at 4 °C and once for 16,500× *g* for 20 min (Beckman Coulter Avanti-J-301 centrifuge, JA-30.50 Ti rotor), the supernatant was filtered through filter 0.22 microns. The filtered supernatant was ultracentrifuged at 100,000× *g* for 2 h. After the first centrifugation, the pellet was resuspended in 8 mL of TBS. The resuspended pellet was ultracentrifuged twice at 100,000× *g* for 2 h (Beckman L8-M centrifuge, SW-60 rotor (Brea, CA, USA). The precipitate was resuspended, filtered through a filter (0.1 µm) and used for additional purification. For additional purification of exosomes, gel filtration on columns with Sepharose 4B was used.

### 4.4. Purification of Vesicle Preparations by Gel Filtration

For additional purification of exosomes, gel filtration on columns with Sepharose 4B separating proteins with molecular weights of 60–20,000 kDa was used as in [38]. The concentrated exosome solutions (0.5 mL) were applied on a column with Sepharose 4B (volume 50 mL) equilibrated in TBS buffer (20 mM Tris HCl (pH 7.5) and 0.5 M NaCl) using a GE Akta Purifier chromatograph (Chicago, IL, USA) and fractions (1 mL) eluted by the same buffer were collected. The exosomes and proteins were monitored by absorbance at 280 nm. For removing of NaCl, the fractions were dialyzed against 20 mM Tris-HCl (pH 7.5) for 14 h at 4 °C and then they were used for different types of analysis. All experiments were carried out under sterile conditions.

### 4.5. Electron-Microscopic Studies of Exosomes

A copper grid covered with formvar film was placed on a drop of the exosome preparations for 1 min, and then the excess liquid was collected with filter paper, and a grid was contrasted with a 0.5% solution of uranyl acetate or 2% solution of phosphorotungic acid for 10–15 s as in [38]. Grids were examined using a Jem1400 (Jeol, Tokyo, Japan) transmission electron microscope supplied with Veleta digital camera (EM SIS, Muenster, Germany). To identify specific markers of exosomes, the vesicles were incubated with mouse monoclonal antibodies against CD81 and CD63 at room temperature for 18 h on a shaker; then, they were sorbed onto grids. Next, the grids were washed with PBS (10 mM Na_2_HPO_4_, 1.76 mM KH_2_PO_4_ (pH 7.4), 137 mM NaCl and 2.7 mM KCl) and incubated with protein A conjugated with 10–12 nm gold nanoparticles for 2 h at room temperature in a humid chamber. Then grids were washed with PBS to remove unbound antibodies, and negatively stained with 2% phosphorotungstic acid solution for 10–15 s. The samples were studied in a Jem1400 electron microscope.

### 4.6. Flow Cytometry of Exosomes

Exosome preparations after two stages of ultracentrifugation and gel filtration were incubated with latex balls (Invitrogen, Waltham, MA, USA) with aldehyde-sulphate groups for 30 min at room temperature. TBS buffer (pH 7.5; (20 mM Tris-HCl, pH 7.5, 0.15 M NaCl)) was added to a final volume of 200 μL, and the mixture was incubated overnight at 4 °C with stirring. Next, the mixtures with balls were incubated with 1 M glycine (1:1) for 30 min at 22 °C to block unbound aldehyde-sulphate groups, centrifuged at 1600× *g* for 3 min and the precipitate was washed twice using 200 μL of 0.5% bovine serum albumin in 10% fetal bovine serum. Then, samples were incubated with antibodies to CD81 conjugated with allophococyanin fluorescent label, while antibodies to CD9 were conjugated with fluorescein isothiocyanate fluorescent label (Biolegend, San Diego, CA, USA) for 1 h at 4 °C, centrifuged at 1600× *g* for 3 min at 22 °C and the supernatant was removed, the pellet was resuspended in 200 μL TBS. In all cases, the final mixtures corresponded to the same amount of initial solution of the exosomes after the first ultracentrifugation. Latex balls incubated with antibodies in the absence of vesicles were used as negative controls. The obtained samples were analyzed using a FACS Canto II flow cytometer (BD Biosciences, San Jose, CA, USA); the results were processed using the FACSDiva Version 6.1.3 software (BD Biosciences, San Jose, CA, USA). 

### 4.7. Nanoparticle Tracking Analysis of Exosomes

After gel filtration, aliquots of placental vesicle preparations were resuspended in TBS buffer. The relative sizes and content of vesicles of various sizes were evaluated by analysis of the trajectory of their movement (Nanoparticle tracking analysis) using device Nanosight NS300 (Malvern, the UK). For each preparation, 3 consecutive surveys of length 10 s each were performed. Data was analyzed using Nanosight NTA v3.2 (Malvern Instruments, Malvern, United Kingdom).

### 4.8. SDS-PAGE Assay

Electrophoretic analysis of exosome proteins was performed according to the Laemmli method using 4–18% polyacrylamide gel containing 0.1% SDS [38]. Exosome preparations containing 20–45 µg of proteins before SDS-PAGE were preincubated in buffer A containing 50 mM Tris-HCl, pH 6.8, 1% SDS, 10% glycerol, 0.025% bromophenol blue, 10 mM EDTA, with or without 10 mM DTT, at 100 °C for 10–15 min, and then applied to the gel. In some cases, before the electrophoresis, the vesicles were preincubated in the presence of trypsin (10 μM, Promega, Fitchburg, WI, USA) or chymotrypsin (5 μm, Sigma) for 5 min at 37 °C in a mixture containing 25 mM Tris-HCl, pH 7.5, 0.33 mM DTT, 0.03 mM EDTA, 1.3 mM NaCl, 2.5 mM MgCl_2_. The reaction was stopped by adding buffer A, and then the proteins were separated by SDS-PAGE. Electrophoresis was carried out for 1.5–2 h at 25 °C in buffer: 25 mM Tris-glycine, pH 8.3, 0.1% SDS at 100–170 V. Proteins were stained with Coomassie R-250 or colloidal silver.

### 4.9. Trypsinolysis of Proteins after Electrophoresis

Protein identification was performed using MALDI-TOF MS and MS/MS spectrometry of tryptic hydrolysates after 1D or 2D electrophoresis as in [38]. In case of 2D electrophoresis, separation of proteins was first carried out using a device for isoelectric focusing of proteins (Protean IEF Cell, Bio-Rad, Hercules, CA, USA). To separate proteins in the gel according to their isoelectric points, a strip was used (linear pH 3-10, 18 cm, Bio-Rad, USA). Exosomes were kept in a rehydration buffer (8 M urea, 2% NP-40, 0.2% ampholytes (pH 3–10) and 50 mM DTT) and transferred to the focusing chamber, the strip was placed on top of gel, and mineral oil was applied. The strip was rehydrated passively for 1 h, then actively for 12 h at 50 V. Isoelectrofocusing was performed at 250 V for 15 min, then for 7 h at 10,000 V. After isoelectrofocusing, the strip was incubated for 30 min in buffer containing 0.38 M Tris-HCl pH 8.8, 6 M urea, 20% glycerol, 2% SDS, and 0.001% bromophenol blue. Next, the strip was incubated in the same buffer containing 100 mM iodoacetamide without DTT for 30 min. After incubation, the strip was placed in a gel and SDS-electrophoresis was performed; proteins were stained with Coomassie R-250.

After SDS-PAGE, gels obtained were stained with Coomassie R-250 and their fragments were washed twice with 100 μL milliQ water on a shaker for 15 min, and then twice for 30 min with 50 μL of 50 mM NH_4_HCO_3_ containing 50% acetonitrile. To remove H_2_O, the gel fragments were washed with 100 μL of 100% CH_3_CN for 20 min. Then, these gel fragments were dried for 10 min at 30 °C using a vacuum evaporator. To hydrolyze proteins, 20 µL of 25 mM NH_4_HCO_3_ containing 12.5 µg/mL trypsin for sequencing (Promega) was added to gel pieces, and, after mixture incubation for 45 min at 0 °C, the solution was removed. Next, gel fragments were additionally incubated in 20 μL of 25 mM NH_4_HCO_3_ for 18 h at 37 °C, and the solution was removed. To extract peptides, gel fragments were washed twice with 25 μL of 50 mM NH_4_HCO_3_ containing 50% acetonitrile, on a shaker for 15 min. The fractions obtained after three treatments of gel were combined, lyophilized, dissolved in 10–20 μL of water and used for MALDI-TOF mass spectrometry analysis.

## Figures and Tables

**Table 1:** Data on identification by MS and MS/MS methods of proteins corresponding to the first peak after gel filtration of exo-1 (Figure 5B) and exo-3 (Figure 6A) and their following separation by SDS-PAGE.

Protein Number		Database MM (Da) ^a^	Identified Proteins ^b^
exo-1	exo-3		
1	1	15988 or 15248	Hemoglobin subunit alpha and beta
2		20007	Ferritin ^c^
3	2	25673	CD63
4	3	25809	CD81
	4	35914	Annexin A5
	5	38580	Annexin A2
	6	41710 or 41766	Actin cytoplasmic
5	7	57341 or 57917	Alkaline phosphatase
6	8	69321	HAS ^c^
	9	77014	Serotransferrin
7	10	104788	Alpha-actinin-4
8	11	150–170 kDa	IgG H and L chains ^c^

^a^ MS—determination of a set of peptides from tryptic hydrolysates by MS/MS in accordance with the sequences of peptides (from three to seven peptides). ^b^ For the identification of proteins and their molecular masses, the 2016 SwissProt program was used. ^c^ Unlike other proteins, ferritin, HSA, and IgGs are absent in exosome preparations after their trypsin treatment; apparently, these proteins are not internal exosome proteins.

**Table 2:** Data on identification by MS and MS/MS methods of proteins corresponding to the first peak after gel filtration of exo-3 (Figure 1C) and exo-3 (Figure 6B) and their following separation by 2D electrophoresis.

Protein Number	Numbers of Protein Spots ^a^	Database MM (Da) ^b^	Identified Proteins ^b^
1	1	15988 or 15248	Hemoglobin subunit alpha and beta
2	2, 3, 4, 28	38580	Annexin A2
3	5, 6, 7	35914	Annexin A5
4	8	38690	Annexin A1
5	9, 10, 11, 12, 13,14,15	41710 or 41766	Actin cytoplasmic
6	16,17, 18	150-170 kDa	IgG light chains ^c^
			IgG heavychains ^c^
7	19, 20, 21, 22, 23	57341 or 57917	Alkaline phosphatase
8	24, 25	69321	HAS ^c^
9	26, 27	77014	Serotransferrin

^a^ MS—determination of the set of peptides was performed using tryptic hydrolysates, the structure of the peptides was confirmed using their MS/MS spectra; protein identification was carried out using sequences from three to seven peptides. ^b^ For the identification of proteins and their molecular masses, the 2016 SwissProt program was used. ^c^ Unlike other proteins, ferritin, HSA, and IgGs are absent in exosome preparations after their trypsin treatment; apparently, these proteins are not internal exosome proteins.

## Supplementary Materials

The following are available online at https://www.mdpi.com/1422-0067/20/10/2434/s1.

## Abbreviations

Ab	antibody
Abz	abzyme;
AI	autoimmune
2D-electrophoresis	Two-dimensional electrophoresis (isoelectrophocusing and SDS-PAGE
Evs	extracellular vesicles
MALDI-TOF	matrix-assisted laser desorption/ionization time-of-flight mass spectrometry
MBP	myelin basic protein
MM	molecular mass
MS	multiple sclerosis
SDS-PAGE	sodium dodecyl sulphate–polyacrylamide gel electrophoresis
SLE	systemic lupus erythematosus
