# Supplementary material for: Extra Purified Exosomes from Human Placenta Contain an Unpredictable Small Number of Different Major Proteins
Source: Int J Mol Sci. 2019 May 16;20(10):2434. doi: 10.3390/ijms20102434 (PMC6566543; doi:10.3390/ijms20102434)
Supplement: Supplementary file 1 [file ijms-20-02434-s001.pdf]

Supplementary Table 1. Summarized data of identification of major proteins of placenta exosomes (first peak after gel filtration) using MALDI mass spectrometry of proteins hydrolyzates after 2D-electrophoresis (Fig. 5B) and after SDS-PAGE of four different preparations (Figs 4A and 5A)

Bands and sports used for proteins identification	Identified proteins	Mr(exper.)	Mr(calc)	Score	Uni-que (U)	Peptide subjected to MS/MS[a]
**Analysis of proteins after 2D-electrophoresis**						
Protein spot: 1	Hemoglobin alpha-subunit	1528.63<br>1832.74<br>2995.17	1528.73<br>1832.88<br>2995.48	174>40	U 	K.VGAHAGEYGAEALER.M<br>K.TYFPHFDLSHGSAQVK.G<br>K.VADALTNAVAHVDDMPNALSALSDL HAHK.L
	Hemoglobin beta-subunit	1125.67<br>1273.85<br>1377.82<br>1669.03	1125.57<br>1273.72<br>1377.69<br>1668.88	132>40	U<br><br>U<br>U	K.LHVDPENFR.L<br>R.LLVVYPWTQR.F<br>K.EFTPPVQAAYQK.V<br>K.VLGAFSDGLAHLDNLK.
Protein spots: 2, 3, and 4	Annexin A2	1034.63<br>1085.59<br>1110.67<br>1224.70<br>1243.75<br>1420.83<br>1459.83<br>1542.01<br>1908.09	1034.52<br>1085.67<br>1110.55<br>1224.56<br>1243.62<br>1420.69<br>1459.67<br>1541.84<br>1907.9	110>40	 <br>U<br><br><br>U<br><br><br>U	K.WISIMTER.S<br>K.VLIRIMVSR.S<br>R.QDIAFAYQR.R<br>K.DIISDTSGDFR.K<br>R.TNQELQEINR.V<br>K.SLYYYIQQDTK.G<br>K.SYSPYDMLESIR.K<br>K.GVDEVTIVNILTNR.S<br>R.AEDGSVIDYELIDQDAR.D
Protein spots: 5, 6, and 7	Annexin A5	953.69<br>1000.77<br>1013.67<br>1051.64<br>1105.78<br>1119.19<br>1126.89<br>1154.62<br>1801.91	953.53<br>1000.59<br>1013.51<br>1051.55<br>1105.58<br>1119.59<br>1126.69<br>1154.60<br>1801.86	121>40	 <br><br><br>U<br><br><br>U<br>U	K.FITIFGTR.S<br>K.VLTEIIASR.T<br>R.LYDAYELK.H<br>K.SELTGKFEK.L + Methyl (C-term)<br>R.SEIDLFNIR.K<br>R.SEIDLFNIR.K + Methyl (C-term)<br>K.LIVALMKPSR.L<br>K.GAGTDDHTLIR.V<br>K.YMTISGFQIEETIDR.E
Protein spot: 8	Annexin A1	1261.65<br>1370.71<br>1549.87<br>1738.79	1261.59<br>1370.76<br>1549.81<br>1738.73	51>40	 U<br><br>U	K.TPAQFDADELR.A<br>K.VLDLELKGDIEK.C<br>K.GTDVNVFNTILTTR.S<br>R.SEDFGVNEDLADSDAR.A
Protein spots: 9, 10, 11, 12, 13,14, and 15	Actin, cytoplasmic	 1197.89<br>1514.97<br>1790.14<br>1953.33<br>2214.39<br>2342.49<br>3183.04	 1197.69<br>1514.74<br>1789.88<br>1953.06<br>2214.06<br>2342.16<br>3182.61	196>40	U<br>U<br><br>U<br>U<br>U<br>U	R.AVFPSIVGRPR.H<br>K.IWHHTFYNELR.V<br>K.SYELPDGQVITIGNER.F<br>R.VAPEEHPVLLTEAPLNPK.A<br>K.DLYANTVLSGGTTMYPGIADR.M<br>R.KDLYANTVLSGGTTMYPGIADR.M<br>R.TTGIVMDSGDGVTHTVPIYEGYALPH AILR.L
Protein spots: 16, 17, and 18	Heavy chains of IgGs[b]	1790.00<br>1028.64<br>1773.13<br>1599.89	1789.93<br>1028.58<br>1773.00<br>1599.77	96>40	U 	K.VSVFVPPRDGFFGNPR.K<br>R.QIQVSWLR.E<br>K.GVALHRPDVYLLPPAR.E<br>K.YVTSAPMPEPQAPGR.Y
Protein spots: 19, 20, 21, 22, and 23	Alkaline phosphatase, placental type	1086.63<br>1127.72<br>1304.84<br>1450.82<br>1623.97<br>1950.89<br>2368.39<br>3233.91	1086.49<br>1127.58<br>1304.68<br>1450.65<br>1623.78<br>1950.86<br>2368.13<br>3233.56	308>40	U<br>U<br>U<br>U<br>U<br>U<br>U<br>U	R.IDHGHHESR.A<br>R.GFFLFVEGGR.I<br>K.GNFQTIGLSAAAR.F<br>R.NWYSDADVPASAR.Q<br>R.ALTETIMFDDAIER.A<br>K.DGARPDVTESESGSPEYR.Q<br>R.QQSAVPLDEETHAGEDVAVFAR.G<br>R.AGQLTSEEDTLSLVTADHSHVFSFGGY PLR.G
Protein spots: 24 and 25	Human serum albumin[b]	1623.05<br>1742.16<br>1853.2<br>1874.30<br>1898.29<br>2044.39<br>2202.32	1622.78<br>1741.87<br>1852.9<br>1874.01<br>1897.99<br>2044.09<br>2201.99	190>40	U<br>U<br>U<br>U<br>U<br>U<br>U	K.DVFLGMFLYEYAR.R<br>R.HPYFYAPELLFFAK.R<br>R.RPCFSALEVDETYVPK.E<br>K.SLHTLFGDKLCTVATLR.E<br>R.RHPYFYAPELLFFAK.R<br>K.VFDEFKPLVEEPQNLIK.Q<br>K.EFNAETFTFHADICTLSEK.E
Protein spots: 26 and 27	Serotransferrin	1208.49<br>1262.58<br>1282.49<br>1477.66<br>1702.78	1208.56<br>1262.61<br>1282.56<br>1477.72<br>1702.75	200>40		K.DSGFQMNQLR.G +Methyl(C-term)<br>K.SASDLTWDNLK.G +Methyl(C-term)<br>K.EGYYGYTGAFR.C<br>K.SASDLTWDNLK.G +Methyl(C-term)<br>R.EGTCPEAPTDECKPVK.W

**Identification of additional proteins from their analysis after 1D electrophoresis**						
**Exo-1, spot 2, Fig. 4**	**Light chain of ferritin[b]**	1192.44<br>1490.58<br>1590.73<br>1606.65<br>1718.90	1192.57<br>1490.71<br>1590.88<br>1606.79<br>1718.98	80>40		K.MGDHLTNLHR.L<br>R.DDVALEGVSHFFR.E<br>K.LNQALLDLHALGSAR.T<br>R.LGGPEAGLGEYLFER.L<br>K.KLNQALLDLHALGSAR.T
	**Heavy chain of ferritin[b]**	1152.76<br>1344.84<br>1544.94<br>3278.51	1152.59<br>1344.62<br>1544.70<br>3278.58	75>40		K.ELGDHVTNLR.K<br>K.YFLHQSHEER.E<br>R.QNYHQDSEAAINR.Q<br>R.QINLELYASYVYLSMSYYFDRDDVAL K.N
**Exo-1, spot 3, Fig. 4 and spot 3, Fig. 5**	**CD63**	693.27<br>1100.41<br>1231.40<br>1983.79	693.28<br>1100.46<br>1231.43<br>1983.83	7<br>79>40	U<br>U	MAVEGGM.K<br>K.VMSEFNNNF.R<br>K.CCGAANYTDWE.K<br>R.VPDSCCIDVTVGCGINFNE.K
**Exo-1, spot 4, Fig.4 and spot 3, Fig. 5**	**CD81**	445.28<br>630.29<br>695.30<br>2252.03	445.22<br>630.21<br>695.26<br>2252.03	80>40	U<br>U	K.DQIA.K.<br>K.EDCHQ.K<br>MGVEGCT.K<br>K.QFYDQALQQAVVDDDANNA.K
**Exo-1, spot 7, Fig. 4 and spot 10, Fig. 5**	**Alpha actin 4**	949.51<br>1010.58<br>1028.63<br>1091.67<br>1214.76<br>1225.76<br>1298.78<br>1300.72<br>1351.75<br>1420.83<br>1428.89<br>1506.84<br>1624.88	949.43<br>1010.49<br>1028.53<br>1091.56<br>1214.67<br>1225.66<br>1298.66<br>1300.60<br>1351.62<br>1420.70<br>1428.76<br>1506.70<br>1624.72	121>40		R.QGEAEFNR.I<br>K.QQSNEHLR.R<br>R.TIPWLEDR.V<br>K.NFITAEELR.R<br>K.LASDLLEWIR.R<br>K.DGLAFNALIHR.H<br>R.HRPELIEYDK.L<br>K.HTNYTMEHIR.V<br>K.GISQEQMQEFR.A K.<br>GYEEWLLNEIR.R<br>R.TINEVENQILTR.D<br>K.AGTQIENIDEDFR.D<br>K.HEAFESDLAAHQDR.V

[a]MS - determination of a set of peptides from tryptic hydrolysates, MS/MS in accordance with the sequences of peptides (from three to thirteen peptides).

[b]IgGs, HSA and ferritin, unlike other proteins, are not the internal proteins of the exosomes.
